# Oncostatin M enhances the lengthening of sensory nerves and skin hypersensitivity

**DOI:** 10.3389/fimmu.2025.1571120

**Published:** 2025-07-03

**Authors:** Chie Ishikawa, Ryo Saito, Masataka Suehiro, Kaori Ishii, Yuhki Yanase, Tomoko Kawaguchi, Kazue Uchida, Nozomi Yanagida, Tomofumi Numata, Wataru Sasaki, Rina Kamigaki, Sachio Takeno, Akio Tanaka

**Affiliations:** ^1^ Department of Dermatology, Institute of Biomedical and Health Sciences, Hiroshima University, Hiroshima, Japan; ^2^ Department of Otorhinolaryngology, Head and Neck Surgery, Institute of Biomedical and Health Sciences, Hiroshima University, Hiroshima, Japan; ^3^ Department of Pharmacotherapy, Graduate School of Biomedical and Health Sciences, Hiroshima University, Hiroshima, Japan

**Keywords:** atopic dermatitis, nerve elongation, oncostatin M, pruritus, sensory nerve, itch

## Abstract

**Background:**

Oncostatin M (OSM) is a cytokine that mediates inflammatory processes and is overexpressed in skin lesions of atopic dermatitis (AD). By amplifying neural responses to chemicals such as histamine, OSM increases sensitivity to pruritus. However, the morphological effects of OSM on peripheral sensory nerves and their subsequent impact on pruritus remain unclear. This study investigated OSM-induced peripheral nerve elongation, which may contribute to skin hypersensitivity.

**Methods:**

We assessed neurite outgrowth using primary mouse dorsal root ganglion (DRG) cells treated with OSM, IL-31, or nerve growth factor. Next, we pre-treated the cells with inhibitors of downstream signaling pathways of OSM, including extracellular signal-regulated kinase (ERK), signal transducers and activator of transcription (STAT) 3, c-Jun N-terminal kinase (JNK), and p38, followed by OSM administration to measure neurite outgrowth. Furthermore, OSM receptor β-overexpressing cell lines were established by gene transfer into the DRG cell line, and nerve elongation was measured after OSM administration. *In vivo* studies involved OSM administration in mouse skin models. Immunofluorescence staining was used to evaluate nerve elongation. We examined whether OSM-infused mice had increased hypersensitivity to mechanical stimuli-induced pruritus. Various cytokine stimuli were applied to CD4+ T cells isolated from healthy humans to examine the conditions under which OSM production increases.

**Results:**

OSM significantly induced neurite outgrowth in DRG cells and the effect of OSM surpassed the effects of IL-31 and nerve growth factor. The neurite outgrowth effect of OSM involved the JAK/STAT3, MEK/ERK, and p38/MAPK pathways. Compared to control cells, DRG cell lines that overexpressed OSM receptor β showed significantly enhanced neurite outgrowth upon OSM treatment. *In vivo*, OSM treatment increased nerve elongation in the mouse dermis. Behavioral assays in mice showed that OSM administration increased sensitivity to mechanical stimuli. IL-4 and TNFα increased OSM production in CD4+ T cells.

**Conclusion:**

OSM induces neurite elongation and may contribute to skin hypersensitivity. This suggests the potential utilization of OSM as a therapeutic target for inflammatory skin diseases such as AD.

## 1 Introduction

Atopic dermatitis (AD), a chronic dermatological condition characterized by itchy eczema, severely affects quality of life. Repeated scratching, triggered by persistent intense itching, destroys the skin barrier and allows allergens and other substances to cross the skin barrier, thereby worsening inflammation. Pruritus mediators and regulatory substances secreted from inflammatory cells sustain the itch–scratch cycle, which can be broken by managing persistent itching. Therefore, it is crucial to understand the pruritic mechanisms in AD (1). Studies using skin tissue from patients or animal models of AD, compared with healthy controls, have reported abnormal peripheral sensory nerve density in the epidermis and dermis of AD lesions, whereby these nerve fibers can be activated by external mechanical, chemical, or biological stimuli that trigger pruritus (2). Endogenous pruritogen-induced increase in sensory nerve fiber density suggests a potential contributory effect of elevated nerve density in the severe pruritus of AD (3, 4). Several factors enable the elongation of peripheral sensory nerves in AD such as IL-31, which induces severe pruritus. The IL-31 receptor comprises heterodimers of oncostatin M receptor β (OSMRβ) and IL31RA, and IL-31 induces nerve elongation and branching in sensory nerves, *in vitro* and *in vivo* (5).

Another OSMRβ ligand, the inflammatory cytokine oncostatin M (OSM), is released by monocytes/macrophages, dendritic cells, and T lymphocytes under proinflammatory conditions (6, 7). OSMR is a heterodimer of OSMRβ and gp130, and its downstream signaling is implicated in hematopoiesis, mesenchymal stem cell differentiation, liver regeneration, cardiac remodeling, nociception, inflammation, and metabolism (8). Mutations in the *OSMR* gene, which encodes OSMRβ, are associated with familial localized cutaneous amyloidosis, which is characterized by severe pruritus, and although the underlying mechanism remains unknown, OSMRβ is considered a significant factor in pruritus (9). Recent reports underscore OSM overexpression in skin lesions of AD (9). OSM does not directly induce pruritus, but rather enhances sensitivity to itching by amplifying neural responses to histamine and leukotrienes (10). However, the morphological effects of OSM on peripheral sensory nerves and their subsequent impact on pruritus remain unclear.

In this study, we investigated the effects of OSM on nerve-fiber morphology and the associated changes in skin hypersensitivity.

## 2 Materials and methods

### 2.1 Animals and animal care

All animal experiments were approved by the Institutional Animal Care and Use Committee of Hiroshima University, Hiroshima, Japan, and performed in accordance with the Guidelines for the Care and Use of Laboratory Animals issued by Hiroshima University. Eight-week-old female HR-1 mice were obtained from Charles River Laboratories (Tokyo, Japan).

### 2.2 DRG excision and culture

At 8–12 weeks of age, mice were euthanized with carbon dioxide (CO_2_). Dorsal root ganglion (DRG) extraction and neuronal dissociation were performed as previously reported (9). Dissociated DRG neurons were plated onto poly-L-lysine-coated cell-culture dishes (10 μg/mL, Sigma-Aldrich, US) and cultured overnight at 37°C under 5% CO_2_ in Dulbecco’s Modified Eagle’s Medium F12 with GlutaMax (Gibco ThermoFisher, US), supplemented with 10% fetal bovine serum (Gibco Thermo Fisher, US), 1% penicillin/streptomycin, and 30 ng/mL nerve growth factor (NGF; PeproTech, US). Then, the medium was replaced with fresh medium containing 50 ng/mL NGF, which was removed prior to cytokine stimulation.

### 2.3 Assays for neurite outgrowth

DRG cells were seeded in 96-well plates at a density of 3000 cells/well, cultured in the same medium (described in Section 2.2) but without NGF, and after a 24-h incubation at 37°C under 5% CO_2_, were stimulated with recombinant mouse OSM protein (100 ng/mL; R&D Systems, US), IL-31 (100 ng/mL; Abcam), and NGF (50 ng/mL; PeproTech) for 6–8 days, without changing the media. Neurite outgrowth was assessed using the Neurite Outgrowth Staining Kit (Invitrogen™, Thermo Fisher Scientific, US). Using a microscope (Keyence BZ-X810, Olympus DP73, Japan), 9–11 random fields were imaged per group. Measurements were taken from the edge of the nucleus to the nerve endings using the proprietary software that was provided with the microscope (Keyence BZ-X800 Analyzer 1.1.2.4, Olympus cell Sens Dimension, Japan). The five longest neurites in each field were measured, and the average value was calculated (**Supplementary Figure 1A**). The experiment was repeated in triplicate, and the data were combined for analysis.

### 2.4 Experiments with OSM-signaling inhibition

Inhibition of OSM-induced neurite outgrowth was assessed with four inhibitors (5 μM each): P38 inhibitor SB203580 (Calbiochem, Germany), JNK inhibitor SP600125 (Calbiochem, Germany), ERK inhibitor U0126 (Cell Signaling Technology, US), and STAT3 inhibitor Stattic (Selleck, US). One hour after inhibitor administration, OSM stimulation commenced, and subsequent experiments were conducted as described in Section 2.3.

### 2.5 Induction of OSMRβ-overexpression by gene transfer

Plasmid vectors (OSMRβ: pRP[Exp]-EGFP/Neo-CAG>mOsmr[NM_011019.3], Control: pRP[Exp]-EGFP/Neo-CAG>ORF Stuffer) were purchased from VectorBuilder, US. Plasmids were amplified in *Escherichia coli* DH5α-competent cells, isolated using the QIAprep Spin Miniprep Kit, and visualized by electrophoresis on a 1.5% agarose gel, which was purified using the Endo Free Plasmid Maxi Kit (Qiagen, The Netherlands), and adjusted to 1 μg/μL. The MED17.11, kindly provided by University of Sheffield, is an immortal DRG cell line established from Immortomouse that expresses markers of cells committed to the sensory neuron system (11). It is a simple model for the developmental study of neurite outgrowth and DRG neurons (11). Lipofection-based gene transfer into MED17.11 was performed using Transit-X2 (Mirus Bio, US). Cells were cultured in Dulbecco’s Modified Eagle’s Medium F12, supplemented with 10% fetal bovine serum, 0.1% penicillin/streptomycin, and G418 (neomycin, 1000 µg/mL). A cell line was established using the limiting dilution method, and gene expression was confirmed using qRT-PCR and Western blot (**Supplementary Figures 3A, B**).

### 2.6 Immunofluorescence staining

Immunofluorescence staining was performed on mouse external ear skin and mouse cervical skin as follows: after 1-h fixation in 4% paraformaldehyde at 4°C, tissues were washed with PBS thrice, permeabilized with 0.2% Triton X-100 in PBS for 30 minutes, and blocked for 1 h with 10% heat-inactivated goat serum in PBS. After overnight incubation at 4°C with primary antibodies (Anti-Protein Gene Product 9.5 Antibody, 1:2000 (2.6.1), 1:1000 (2.6.2-3), Millipore, Germany), followed by three washes with PBS, the secondary antibody (Alexa Fluor 750, Abcam, UK; 1:1000), which was added and incubated for 1 h at room temperature. Samples were mounted using ProLong Gold (Thermo Scientific, US) and imaged using a confocal microscope (Keyence BZ-X810, Japan). All mice were 8–12 weeks old and euthanized with CO_2_ inhalation.

#### 2.6.1 External ear skin

Every 3–4 days for 2 months, the mice received OSM (100 ng) or saline subcutaneous pre-injections into the external ear. To assess nerve extension, overall images of the external mouse ear were obtained, followed by random imaging of two peripheral areas per ear; binaural stimulus was used for each mouse. Keyence application software (BZ-X800 Analyzer 1.1.2.4) was used for analysis. A predefined region of interest (ROI) was manually selected to exclude non-tissue areas. Within each ROI, the fluorescent area derived from anti-PGP9.5 immunostaining was binarized and calculated as a percentage of the total area. (**Supplementary Figure 1B**). Three independent research assistants performed the analyses in a blinded manner. For each experiment, ears were obtained from eight mice.

#### 2.6.2 Mouse cervical (rostral back) skin

Every 3–4 days for 2 months, the mice received OSM (100 ng) or saline subcutaneous pre-injections into the rostral back. Cryostat (approximately 20-μm thick) sections of the rostral back skin were fixed in acetone and stained using the procedure described above. Although the same antibody was used as in Section 2.6.1, nonspecific fluorescence signals were occasionally observed in epithelial and vascular tissues in the rostral back skin. To ensure specificity in quantification, regions containing the stratum corneum, hair follicles, and blood vessels were excluded from measurement. Only fluorescent areas located within the dermis and showing morphological features consistent with peripheral nerves (such as linear organization, direction and location) were selected for analysis. Quantification of the fluorescent area was performed using the same method described in Section 2.6.1 with BZ-X800 Analyzer software. Eight mice were used in each group for the experiment, and 2–3 skin sections per mouse were used for measurements.

### 2.7 Measurement of scratching behavior

Itch-related scratching behavior was analyzed using a SCLABA^®^-Real system (Noveltec, Kobe, Japan). Mice received OSM (100 ng) or saline subcutaneous pre-injections in the rostral back every 3–4 days for 1 month. Behavior was monitored remotely for 60 minutes at ambient room temperature (22°C) (12). Seven mice were used in each group.

### 2.8 Mechanical alloknesis assay

Mechanical alloknesis was assessed, with at least seven mice per group, using von Frey filaments (0.16 g bending force) and modified methods (12). Briefly, each mouse received three innocuous filament-based mechanical stimuli on the rostral dorsal surface of at least 5-s intervals (average 20 s). This sequence was repeated 10 times at 3-min intervals (30 times total). Scores were calculated based on the total number and duration of scratching responses. In this study, 0.16g von Frey filaments was used in accordance with previous reports (12).

### 2.9 Purification and culture of human CD4+ T cells

Peripheral blood mononuclear cells (PBMCs) from healthy volunteers were isolated in Leucosep™ tubes (Greiner Bio-One Co, Tokyo. Japan) by density gradient centrifugation with Ficoll (GE Healthcare Japan, Tokyo. Japan). CD4+ T cells were then purified from PBMCs using the Human CD4+ T cells Isolation Kit (STEMCELL Technologies, Tokyo, Japan). CD4+ T cells were cultured in RPMI medium supplemented with 5% human serum at 37°C under a 5% CO_2_ atmosphere for 24 h, then seeded on 24 well plates and treated for 1 h with RPMI medium containing 10 ng/mL of various test substances, including IL-4 (R&D Systems, Minneapolis, MN, USA), IL-13 (R&D Systems), IL-31 (R&D Systems), TSLP (R&D Systems), GM-CSF (R&D Systems), and TNF-α (R&D Systems).

### 2.10 Real-time quantitative PCR

Total RNA was isolated using the RNeasy Mini Kit (Qiagen), according to the manufacturer’s instructions. First-strand cDNA was synthesized from isolated RNA using a QuantiTect^®^ Reverse Transcription Kit (Qiagen), and RT-qPCR was performed using a QuantStudio 3 real-time PCR system (ThermoFisher) with the following thermocycle: denaturation at 95°C for 15 s, annealing at 60°C for 60 s. The expression of the glyceraldehyde 3-phosphate dehydrogenase gene (GAPDH)was measured as the internal control. Primer pairs for detecting OSM, the OSMR, were obtained from ThermoFisher Scientific.

### 2.11 Statistical analysis

Data are presented as the mean ± standard deviation or ± standard error of the mean as indicated. Statistical analyses were performed using GraphPad Prism 9.5.1 (GraphPad Software, US). An independent samples *t*-test was performed for two-group comparisons, and analysis of variance with the Kruskal–Wallis test was performed to compare three or more groups. Fisher’s exact test was performed to compare qualitative data. Statistical significance was set at *p*<0.05.

## 3 Results

### 3.1 OSM induces nerve-fiber elongation

**Figure 1:**
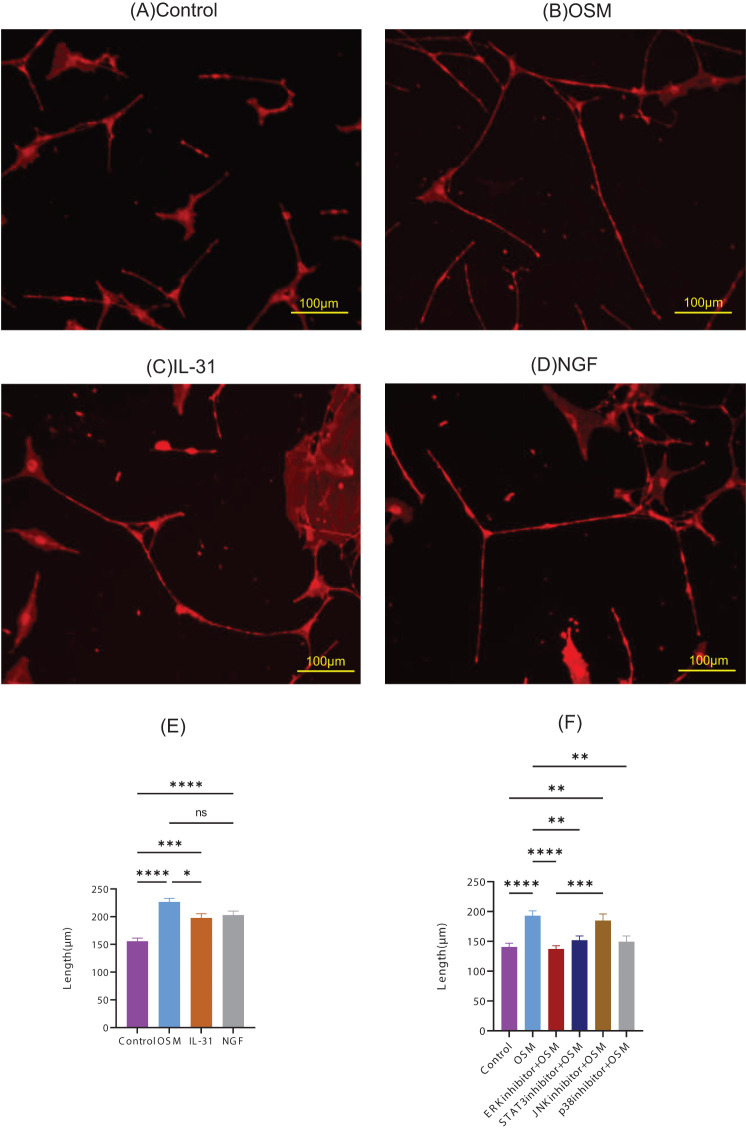
Representative microscopic images of primary DRG cells cultured with the following stimulatory conditions: **(A)** control, **(B)** OSM, **(C)** IL-31, and **(D)** NGF. The neurite length measurements and statistical analyses are presented in **(E)**. The neurite lengths of OSM-stimulated primary DRG cells pretreated with the inhibitors were measured and statistically evaluated **(F)**. For each group, 9–11 random fields were imaged, and the five longest neurites per field were measured. The experiment was repeated in triplicate, and the data were combined for analysis. Data are presented as the mean ± standard error (SE), *p<0.05, **p<0.01, ***p<0.001, ****p<0.0001, ns, not significant; DRG, dorsal root ganglion; OSM, oncostatin M; NGF, nerve growth factor.

To examine OSM-induced morphological changes in peripheral nerve fibers, we cultured primary DRG cells with OSM, IL-31, and NGF, and measured the nerve-fiber length. IL-31 and NGF, which induce nerve growth, were used as positive controls. Nerve-fiber elongation was observed for all stimulations (**Figures 1A–D**), and statistically longer than those in the control group. Compared with IL-31, OSM showed statistically predominant neurite outgrowth (**Figure 1E**). DRG cells showed OSM concentration-dependent neurite outgrowth (**Supplementary Figure 2A**). The OSMRβ expression in the DRG of HR-1 mice was confirmed by qRT-PCR (**Supplementary Figure 2B**).

### 3.2 OSM-induced neuronal outgrowth via multiple intracellular signaling pathways

To identify the signaling pathways involved in OSM-induced nerve elongation, molecules in the downstream signaling pathways of OSM, such as ERK, STAT3, JNK, and p38, were blocked with their respective inhibitors. Primary DRG cells were treated with inhibitors before OSM stimulation. Pretreatment with ERK, STAT3, and p38 inhibitors suppressed neurite outgrowth in OSM-stimulated cells, indicating that JAK/STAT3, MEK/ERK, and p38/MAPK signaling mediate OSM-induced neurite outgrowth. JNK inhibitors did not suppress the nerve elongation effect of OSM (**Figure 1F**).

### 3.3 OSMRβ overexpression enhanced neuronal outgrowth

**Figure 2:**
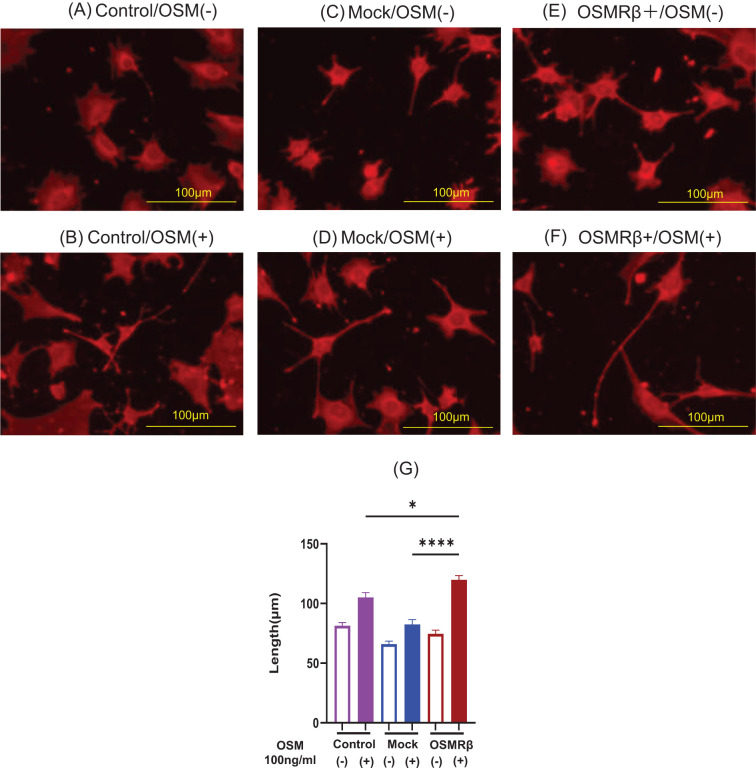
Representative microscopic images of cells cultured with OSM stimulation in Control, Mock, and OSMRβ-overexpressing cell lines. **(A)** Control/OSM-, **(B)** Control/OSM+, **(C)** Mock/OSM-, **(D)** Mock/OSM+, **(E)** OSMRβ overexpressing/OSM-, **(F)** OSMRβ overexpressing/OSM+. Results of neurite length measurements and statistical evaluation after OSM stimulation of Control, Mock, and OSMRβ-overexpressing cell lines **(G)**. Data are presented as the mean ± standard error (SE), *p<0.05, ****p<0.0001. For each condition, 9–11 random fields were imaged, and the five longest neurites per field were measured. The experiment was repeated in triplicate, and the data were combined for analysis. OSM, oncostatin M; OSMR, oncostatin M receptor.

To more reliably investigate the effect of OSM signaling on neurite outgrowth, we performed gene transduction using the DRG cell line (MED17.11) to establish a cell line that stably overexpresses OSMRβ. OSM was administered to OSMRβ-overexpressing, control vector-transfected, and parental cells. OSMRβ-overexpressing DRG cells demonstrated predominant neurite outgrowth upon OSM treatment (**Figures 2A–F**) compared to parental cells and mock cells (**Figure 2G**).

### 3.4 OSM subcutaneous injection induced neuronal outgrowth in mouse skin

**Figure 3:**
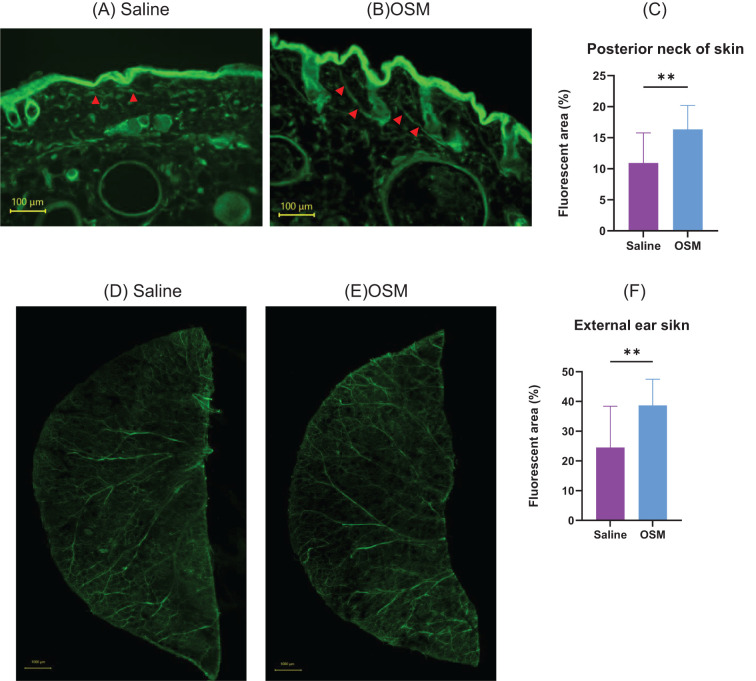
Mice were subcutaneously injected with either saline or OSM in the back of the neck for two months, followed by fluorescent immunostaining. **(A)** Saline and **(B)** OSM. Arrows indicate stained nerve fibers. Statistical evaluation of neurite elongation and enlargement is shown in **(C)**. Eight mice were used in each group, and 2–3 skin sections per mouse were analyzed for quantification. Mice were subcutaneously injected with either saline or OSM in the external ear for two months, followed by fluorescent immunostaining: **(D)** saline and **(E)** OSM. Statistical evaluation of neurite elongation and enlargement is presented in **(F)**. Both ears of each mouse were used for analysis, and two peripheral areas were randomly imaged per ear (i.e., four areas per mouse). A total of eight mice were analyzed per group. Data are presented as the mean ± standard deviation (SD), **p<0.01. OSM, oncostatin M.

Immunofluorescence staining was performed on mouse rostral back skin and external ear skin to examine the effects of OSM on neuronal elongation *in vivo*. As it is easier to evaluate nerve elongation with fewer hair follicles and to perform von Frey filament stimulation in the subsequent experiment in hairless subjects, HR-1 mice were used in this study. Fluorescent immunostaining of mouse rostral back skin showed that in the dermis, the OSM-treated group had a predominant extension of the peripheral nerve (**Figures 3A–C**); *p*=0.0004). Whole-mount immunofluorescence staining of the external ear skin showed that, compared to the saline-treated group, the OSM-treated group had elongated peripheral cutaneous nerves that extended to the limbus (**Figures 3D–F**; *p*<0.0001). No difference was observed in the appearance or thickness of the outer ear skin of mice treated with OSM. Similarly, there was no change in the appearance of the skin of the rostral back (data not shown).

### 3.5 OSM-induced neuronal outgrowth increased mechanical stimulation-induced scratching behavior

**Figure 4:**
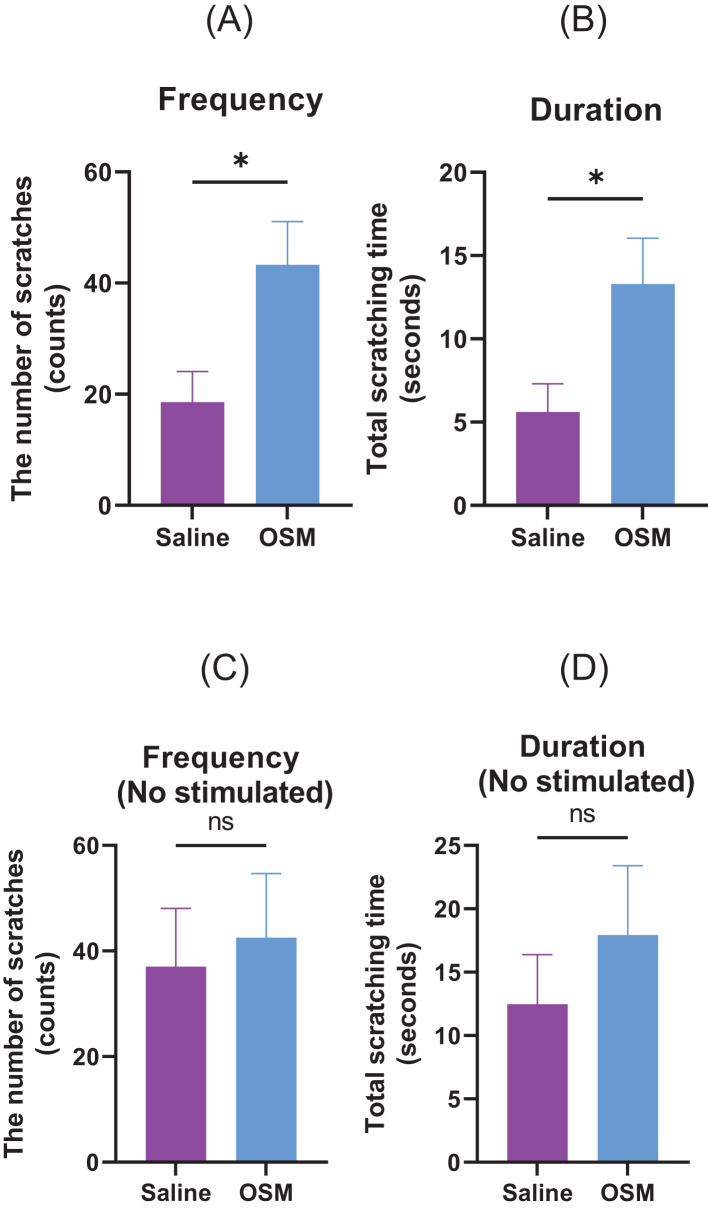
Mice were subcutaneously injected with either saline or OSM in the back of the neck for one month. Scratching behavior in response to von Frey filament stimulation was recorded in terms of **(A)** number of occurrences and **(B)** duration. Those measured without stimulation by von Frey filaments are **(C)** number of occurrences and **(D)** duration. A total of seven mice were used per group (N=7). Data are presented as the mean ± standard error (SE), *p<0.05. OSM, oncostatin M. ns, not significant.

We investigated whether OSM-induced nerve elongation enhanced hypersensitivity to mechanical stimuli-induced pruritic scratching (**Figures 4A, B**). Compared to the control group, the von Frey filament-induced scratching behavior of the hindlimbs significantly increased in the number of scratches and scratching duration in the group receiving long-term OSM administration. In the absence of stimulation with von Frey filaments, there was no significant difference between the control and OSM-treated groups (**Figures 4C, D**). Although OSM administration alone did not alter scratching behavior, long-term OSM administration significantly enhanced hypersensitivity to mechanical stimuli. Similarly, we investigated the effects of histamine and IL-31, both of which are chemical mediators that induce pruritus. No significant differences were observed in scratching behavior in response to histamine regardless of OSM administration. In contrast, mice administered OSM exhibited a slight increase in both scratching frequency and duration following IL-31 injection; however, these differences were not statistically significant in the present study (**Supplementary Figure 4**). Furthermore, we analyzed the duration of scratching bouts using the SCLABA^®^-Real system to distinguish between short-lasting (<1.5 seconds) and long-lasting (≥1.5 seconds) events, according to previous definitions (13). Nearly all scratching bouts in our experiments were classified as short-lasting, with long-lasting events being rare and not statistically analyzable (data not shown).

### 3.6 CD4+ T cells show enhanced production of OSM in the presence of Il-4, TNFa

**Figure 5:**
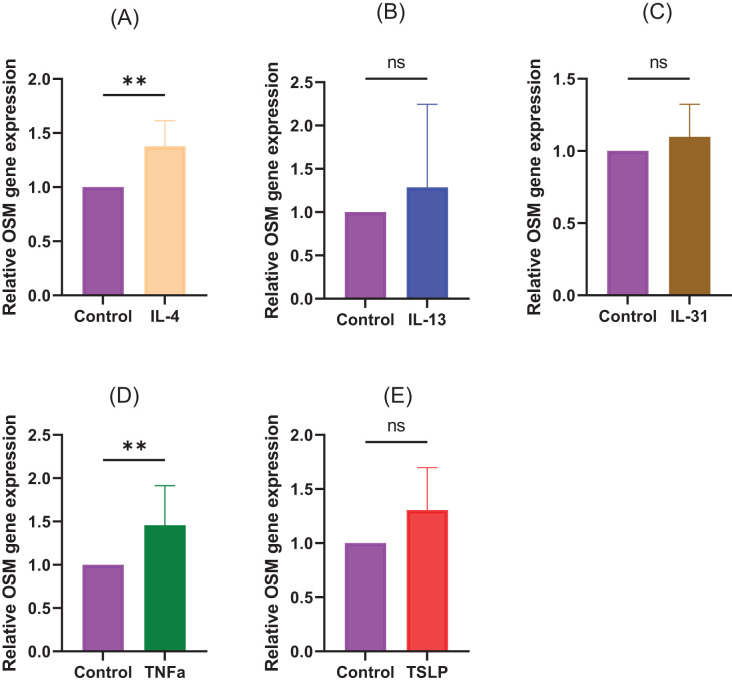
CD4+ T cells isolated from healthy humans were stimulated with **(A)** IL-4 (10 ng/mL), **(B)** IL-13 (10 ng/mL), **(C)** IL-31 (10 ng/mL), **(D)** TNFa (10 ng/mL), and **(E)** TSLP (10 ng/mL). N=8 samples from 8 human volunteers. Data are presented as the mean ± standard deviation (SD), **p<0.01, ns, not significant.

To examine under what conditions the production of OSM is enhanced, CD4+ T cells isolated from healthy humans were stimulated with each cytokine, and then the expression level of OSM was measured by qRT-PCR. The results showed that the expression of OSM was predominantly increased after stimulation with IL-4 and TNF-α. No significant increase was observed for IL-13, IL-31, and TSLP. (**Figure 5**, (A) IL-4: *p*=0.0057, (B) IL-13: *p*>0.9999, (C) IL-31: *p*=0.0831, (D) TNF-α: *p*=0.0057, (E) TSLP: p=0.0831).

## 4 Discussion

This study demonstrated the effect of OSM on elongating peripheral nerve fibers. *In vivo* experiments using mouse skin showed that OSM administration elongated peripheral sensory nerves. In mice treated with OSM, mechanical stimulation-induced itching was enhanced.

Compared to healthy individuals, patients with AD exhibit hypersensitivity to itching that frequently manifests as alloknesis, itching secondary to normally non-itchy stimuli (e.g., friction from clothing), and hyperkinesis, intensified itching from typical pruritic stimuli (1). Histological examination has reported increased peripheral nerve length and density following neuronal activation by external mechanical, chemical, and biological stimuli, such as clothes-induced friction, histamines, and cytokines, in the affected areas of patients with AD and animal models, which may partially account for intense pruritus (2, 4). It has been previously reported that an imbalance between neurotrophins, such as NGF, and repulsive factors, such as Sema3A, is involved in the alteration of peripheral nerve density in such lesions in patients with AD (3). More recently, a role for TH2 cytokines in promoting sensory nerve elongation in AD has also been proposed (14).

IL-31 is a key cytokine that induces pruritus in AD. Produced by activated CD4+ Th2 cells, IL-31 activates the DRG via receptors on C-fiber nerve terminals in the skin to elicit pruritus (5). Elevated IL-31 levels are observed not only in AD but also in other skin conditions, such as prurigo nodularis, psoriasis, and chronic urticaria, and indicate its role as an early pruritogenic mediator in diverse skin diseases (9). Feld et al. reported that IL-31 promotes neurite outgrowth and branching, *in vitro* and *in vivo*, which explains the clinical observation of increased sensitivity to minimal stimuli and persistent itching in AD (5). OSM and IL-31 utilize the OSMRβ chain. Although OSM is upregulated in lesional areas of AD, its specific role remains unclear. We investigated OSM-induced morphological changes in peripheral nerves and examined their effects on alloknesis. Using cultured primary DRG cells from mice, we investigated the effects of OSM on peripheral neuronal morphology, which revealed significantly increased neurite outgrowth in the OSM-treated group compared to the control group. They showed OSM concentration-dependent neurite outgrowth. The OSM-enhanced neurite outgrowth surpassed that seen with IL-31, which is a known stimulator of neurite outgrowth (**Figure 1**). OSM binds to the OSMR-gp130 receptor complex, which activates Jak1 and Jak2 kinases that phosphorylate tyrosine residues in gp130 and OSMR and leads to the recruitment of STAT1 and STAT3, which undergo nuclear translocation to regulate gene transcription. OSM also activates the RAS-MAPK cascade via SHP2 and Shc, which drives ERK1/2, JNK, and p38 activation (15).

Using inhibitors of OSM signaling pathways, we ascertained that the JAK/STAT3, MEK/ERK, and p38/MAPK pathways were crucial for OSM-mediated neurite outgrowth. Recently, JAK inhibitors have emerged as topical or oral therapeutic agents for AD. Our study suggests that OSM-induced neurite outgrowth can be suppressed by inhibiting the JAK/STAT3 pathway and underscores its importance in controlling neurite outgrowth. These new insights facilitate the development of therapies targeted at promoting nerve regeneration and neuroprotection.

After establishing OSMRβ-overexpressing mouse DRG cell lines, we compared them to parental cells and control vector-transduced cells (Mock), and found that OSMRβ-overexpressing cell lines exhibited significantly increased neurite length upon OSM stimulation, which further confirms OSM signaling-induced neurite outgrowth in DRG cells. To investigate whether OSM induces peripheral sensory nerve extension *in vivo*, we used mouse rostral cervical and external ear skin. OSM increased the peripheral sensory nerve density in mouse rostral cervical skin, and the nerves extended further toward the external ear skin periphery, which confirmed the peripheral nerve-elongation effect *in vivo*. We found that OSM promoted the elongation of peripheral sensory nerves and enhanced hypersensitivity to mechanical stimuli. Using a mild stimulus from a von Frey filament in mice, we evaluated alloknesis and hyperknesis. Compared to the saline-treated group, the >1-month OSM-treated group showed significantly increased frequency and duration of scratching behavior but, in the absence of mechanical stimuli, showed no significant intergroup difference in response. In a study by Tseng et al. (10) that used calcium imaging and electrophysiological techniques, unlike other pruritogenic cytokines, OSM did not directly itch-sensitize neurons, but rather enhanced pruritus by potentiating the neural response to pruritogens. We found that OSM administration alone did not alter scratching behavior in mice, but increased hypersensitivity to stimuli with a von Frey filament. This suggests that OSM does not directly induce itching, but rather enhances hypersensitivity to itching through nerve elongation, which enhances the sensory network, lowers the itch threshold, triggers symptoms with minimal scratching stimuli, and is considered one of the main causes of antihistamine-resistant pruritus (5, 16). In addition, we assessed scratching behavior induced by chemical pruritogens such as histamine and IL-31 (**Supplementary Figure 4**). While histamine did not significantly affect scratching behavior regardless of OSM treatment, IL-31 administration led to a slight but non-significant increase in scratching frequency and duration in OSM-treated mice. Notably, previous studies, including Takaoka et al. (13), have reported that IL-31 induces long-lasting scratching (≥1.5 seconds per bout). However, in our experiments, nearly all observed scratching bouts were short-lasting (<1.5 seconds), with very few long-lasting events. This discrepancy may be due, at least in part, to differences in mouse strains, which could influence the threshold or patterns of pruritic behavior.

OSM is released by monocytes/macrophages, dendritic cells, and T lymphocytes (6, 7). In addition, various inflammatory cells, including T lymphocytes, that produce OSM infiltrate the lesions of patients with atopic dermatitis. In the present study, we investigated the conditions for OSM production and found that stimulation with IL-4 and TNFα predominantly increased OSM production in CD4+ T cells isolated from healthy subjects. Moreover, TSLP showed an increasing trend, although no statistically significant difference was observed in this study (**Figure 5**). This study indicates that both type 1 and type 2 inflammation can affect OSM production and may be a source of OSM in AD patients.

Suehiro et al. reported that granulocyte-macrophage colony-stimulating factor and IL-4 enhanced OSM expression in isolated human monocytes, with higher levels of CCL2 expression in the skin lesions of AD and psoriasis vulgaris (9). They reported that when mice were given OSM by tail vein injection a total of four times every 8 h, and DRGs were collected, the expression of receptor subunits of OSMR, IL-4 and IL-13 was promoted and the expression of IL-31ra was suppressed (9).

There have been several reports on increased OSM expression in type 2 inflammation-driven disease. OSM expression significantly increased in nasal polyp tissue from patients with chronic rhinosinusitis with nasal polyps and correlated with IL-13 expression in the sinus mucosa (17). Studies of patients with bronchial asthma revealed prominent nerve elongation in those with eosinophilia-associated bronchial asthma (18) and enhanced OSM signaling in severe asthma (19). These reports suggest that OSM induced by type 2 cytokines such as IL-4 is associated with the pathology of each disease and further research is warranted. Slaets et al. found neurite outgrowth in cultured neuronal cells in the CNS in an OSM capacity-dependent manner and reported that OSM protected primary neurons in culture from cell death. the effects of OSM on nerves may not only affect DRGs but also other neurons (20).

This study had some limitations. As this study only investigated mouse nerves, the effects of OSM on human nerves remain unknown. The effect of inflammation induced by OSM subcutaneous injection on pruritus has not been examined. In this study, we focused on the effects of nerve elongation by administering OSM to mice over a relatively long period. However, changes that may occur with short-term administration should be a subject for future study. In this study, we also experimented with downregulating OSMRβ expression, but this was not feasible owing to the significantly reduced survival rate of DRG cells and the weakened state of the cells. Instead, we performed experiments with cells in which OSMRβ expression was upregulated. In immunofluorescence staining, we used the pan-neuronal marker PGP9.5 to measure nerve elongation in the dermis, but we were not able to distinguish between C fibers (including NP1, NP2, NP3) and other fibers. In addition, although intraepidermal nerve fibers are of particular interest in studies of cutaneous innervation, we were unable to accurately evaluate nerve fibers in the epidermis due to nonspecific fluorescence signals observed in the stratum corneum under the current staining conditions. To ensure the specificity and reliability of our analysis, we therefore restricted our measurements to morphologically identifiable nerve structures within the dermis, excluding the epidermis and other regions such as hair follicles and adipose tissue. This study did not use OSMRβ-deficient mice or disease model mice, and this is an issue for future research.

The anti-OSMR antibody vixarelimab (KPL-716) is undergoing clinical trials for the treatment of prurigo nodularis (ClinicalTrials.gov Identifier: NCT03816891) (21). By binding to the common OSMRβ subunit, it targets both the IL-31 and OSM pathways and has been reported to predominantly improve pruritus (21). Our research suggests that targeting OSM as a therapeutic approach may be effective for pruritus that is refractory to treatment, such as alloknesis. It is essential to continue investigating OSM in future studies.

In conclusion, OSM signaling induces peripheral sensory nerve elongation in the skin, which potentially increases hypersensitivity to mechanical stimuli. Advancing research on the role of OSM in AD could potentially facilitate OSM modulation through novel targeted therapeutic strategies.

## Data Availability

The original contributions presented in the study are included in the article/**Supplementary Material**. Further inquiries can be directed to the corresponding author.
