# Supplementary material for: Oncostatin M enhances the lengthening of sensory nerves and skin hypersensitivity
Source: Front Immunol. 2025 Jul 3;16:1571120. doi: 10.3389/fimmu.2025.1571120 (PMC12267034; doi:10.3389/fimmu.2025.1571120)
Supplement: Supplementary file 1 [file Presentation1.pptx]

## Slide 1
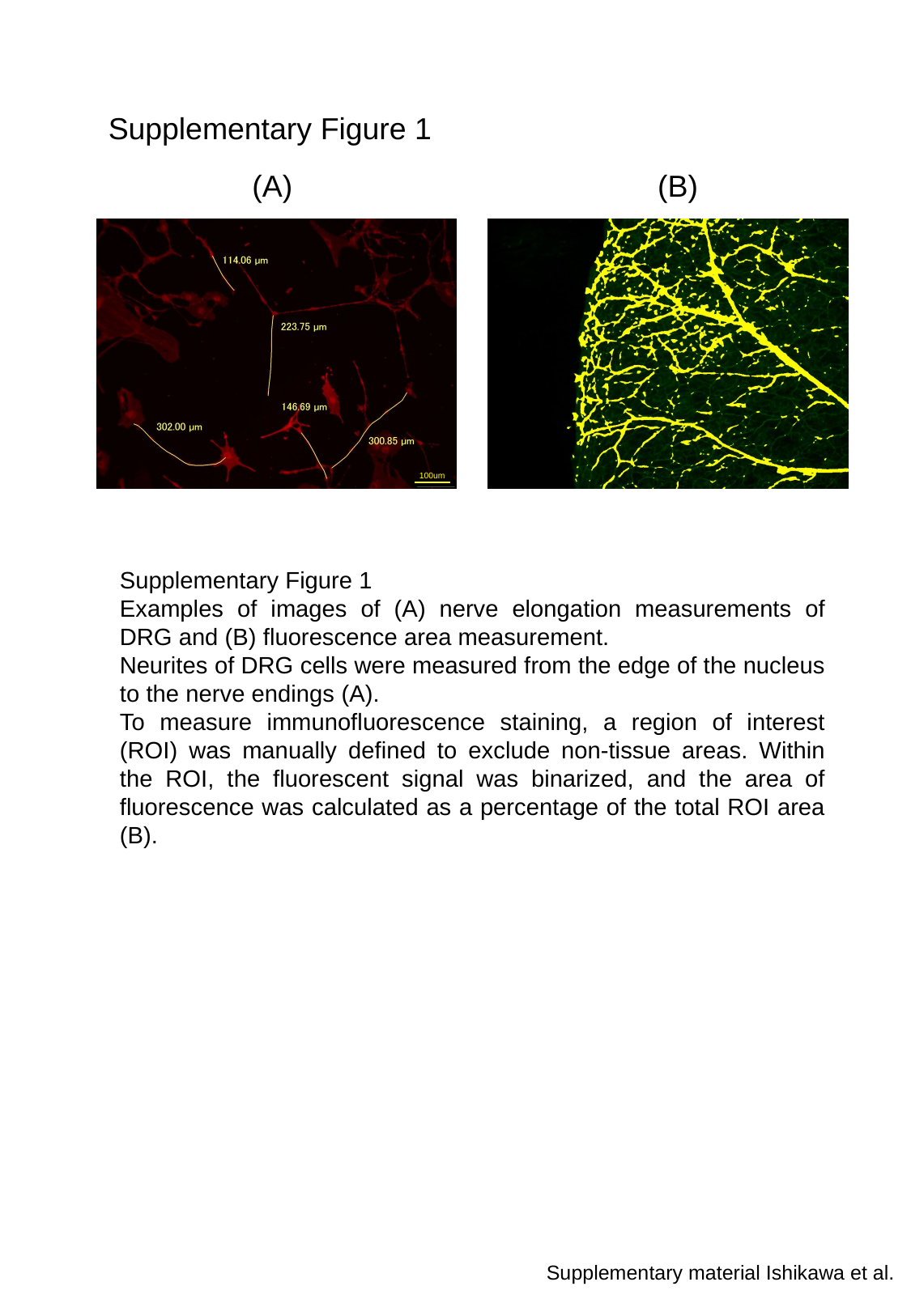

Supplementary Figure 1
(A)
(B)
100um
Supplementary Figure 1
Examples of images of (A) nerve elongation measurements of DRG and (B) fluorescence area measurement.
Neurites of DRG cells were measured from the edge of the nucleus to the nerve endings (A).
To measure immunofluorescence staining, a region of interest (ROI) was manually defined to exclude non-tissue areas. Within the ROI, the fluorescent signal was binarized, and the area of fluorescence was calculated as a percentage of the total ROI area (B).
Supplementary material Ishikawa et al.

## Slide 2
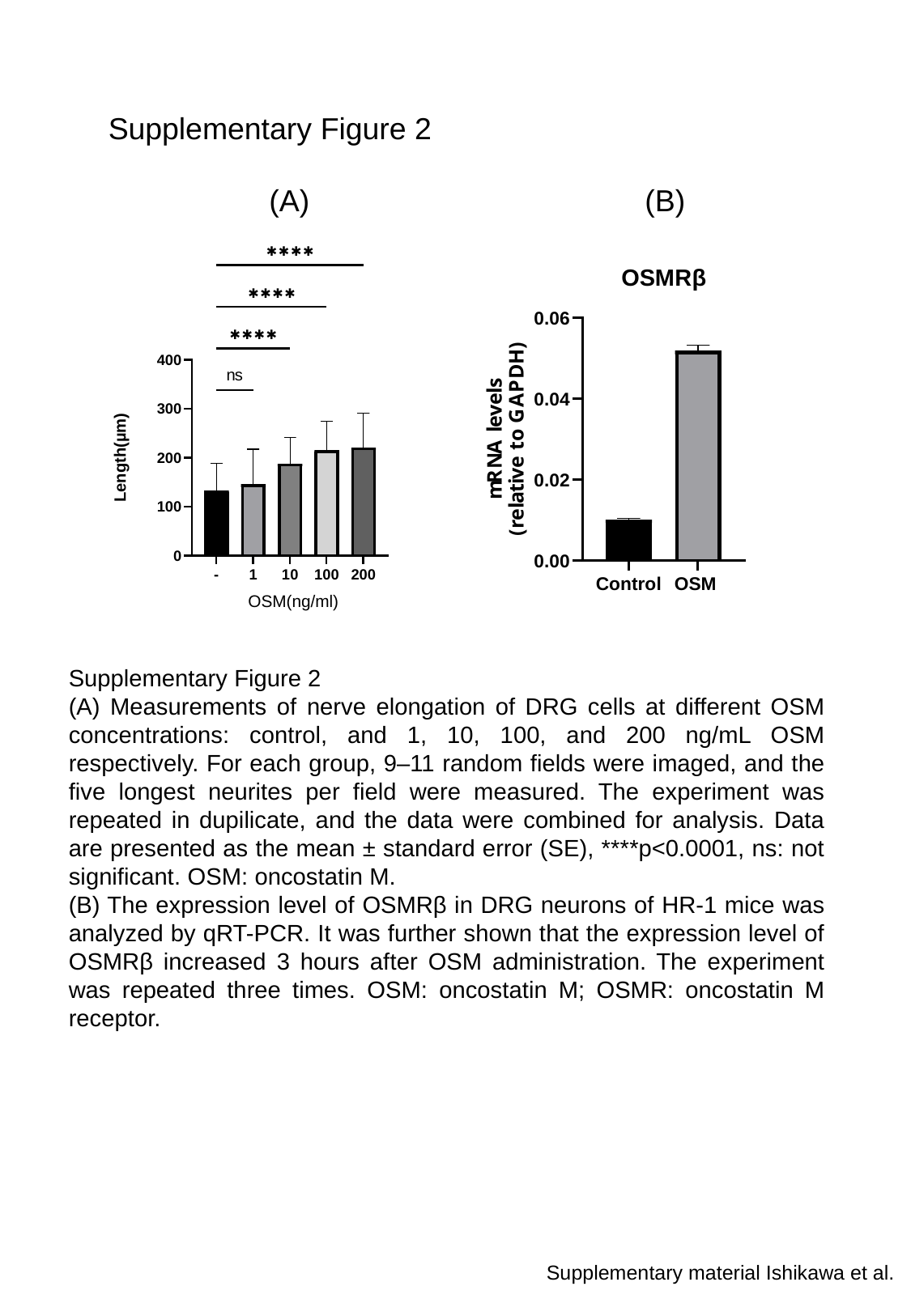

Supplementary Figure 2
(A)
(B)
OSM(ng/ml)
Supplementary Figure 2
(A) Measurements of nerve elongation of DRG cells at different OSM concentrations: control, and 1, 10, 100, and 200 ng/mL OSM respectively. For each group, 9–11 random fields were imaged, and the five longest neurites per field were measured. The experiment was repeated in dupilicate, and the data were combined for analysis. Data are presented as the mean ± standard error (SE), ****p<0.0001, ns: not significant. OSM: oncostatin M.
(B) The expression level of OSMRβ in DRG neurons of HR-1 mice was analyzed by qRT-PCR. It was further shown that the expression level of OSMRβ increased 3 hours after OSM administration. The experiment was repeated three times. OSM: oncostatin M; OSMR: oncostatin M receptor.
Supplementary material Ishikawa et al.

## Slide 3
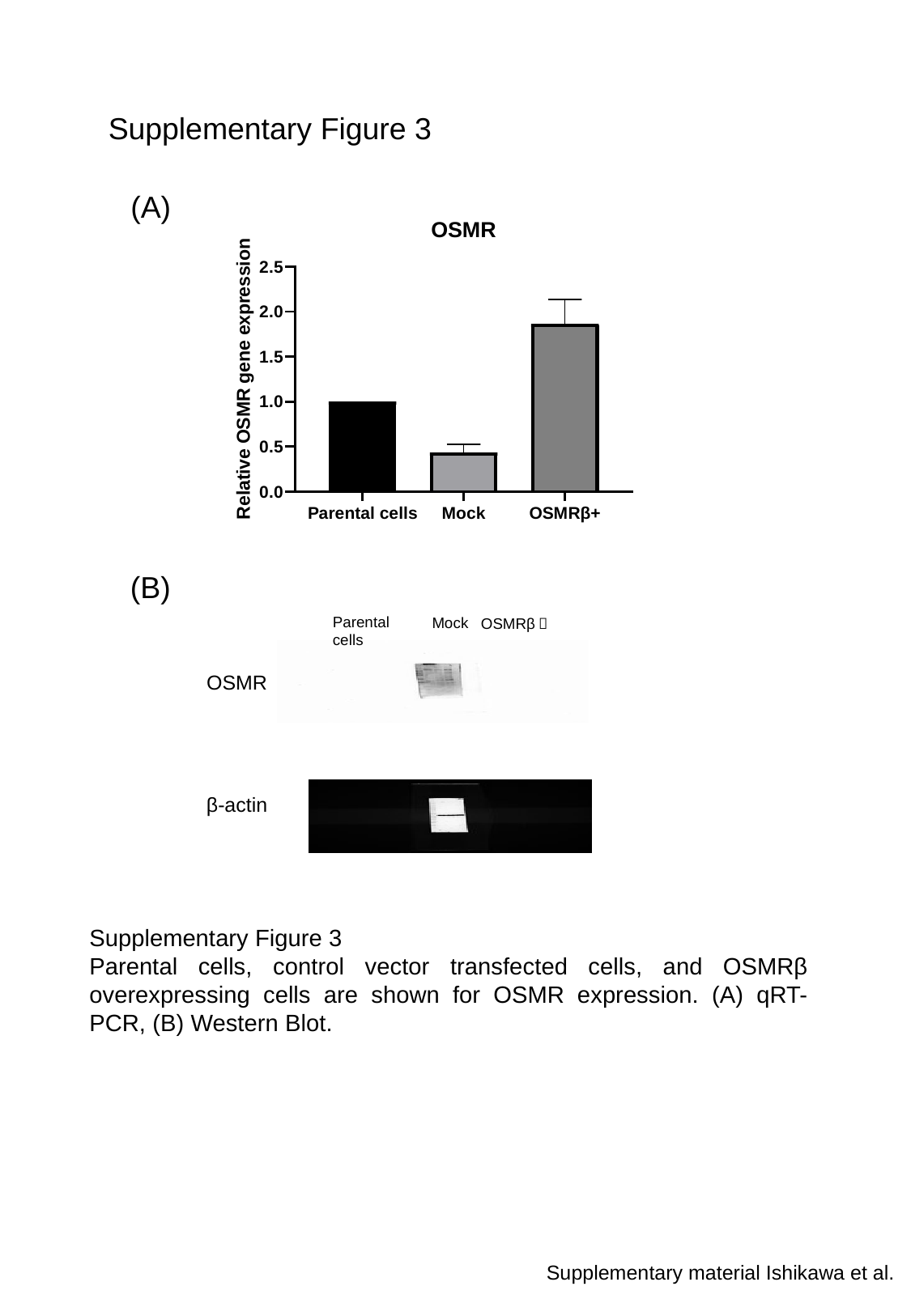

Supplementary Figure 3
(A)
(B)
Parental cells
Mock
OSMRβ＋
OSMR
β-actin
Supplementary Figure 3
Parental cells, control vector transfected cells, and OSMRβ overexpressing cells are shown for OSMR expression. (A) qRT-PCR, (B) Western Blot.
Supplementary material Ishikawa et al.

## Slide 4
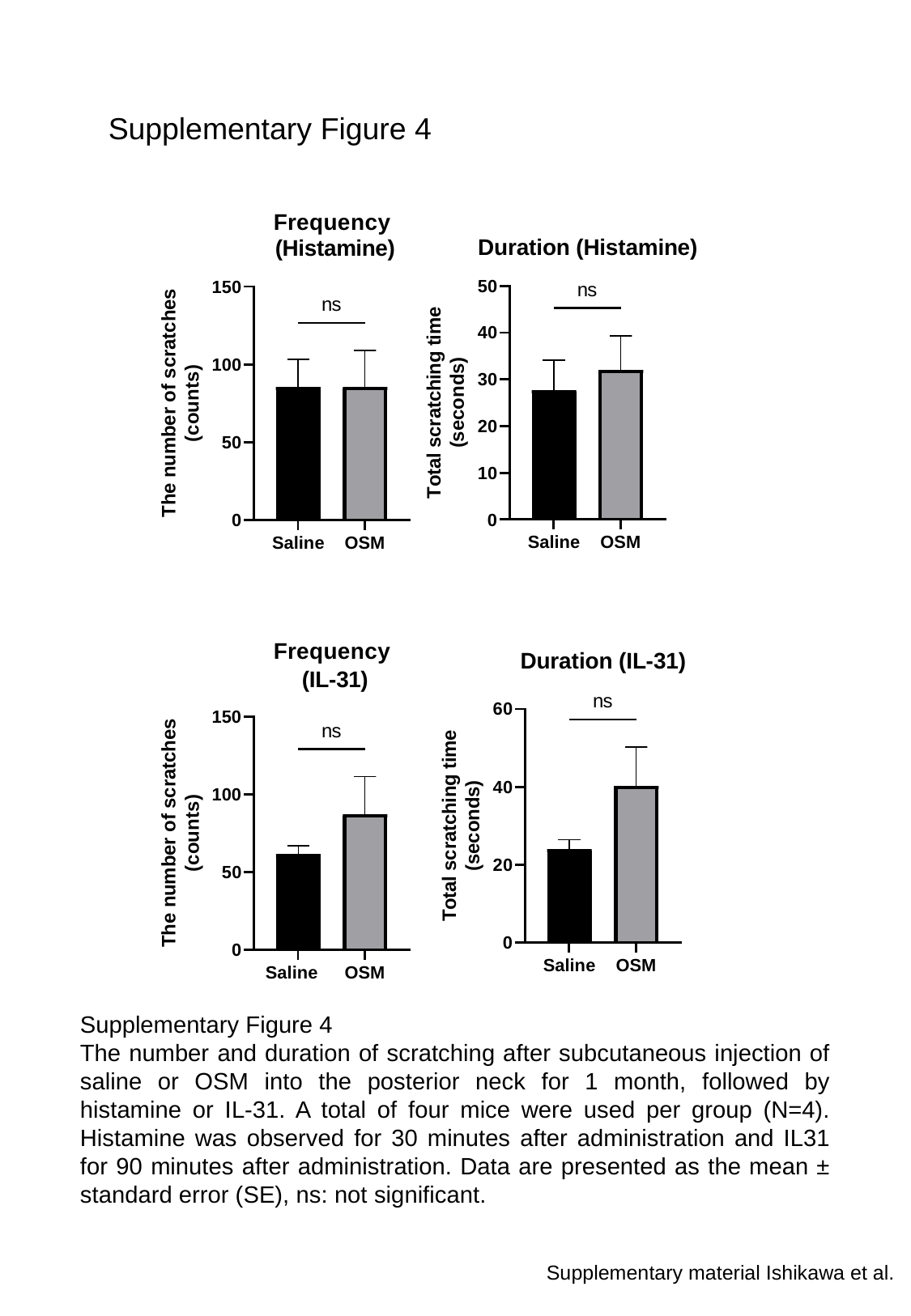

Supplementary Figure 4
Supplementary Figure 4
The number and duration of scratching after subcutaneous injection of saline or OSM into the posterior neck for 1 month, followed by histamine or IL-31. A total of four mice were used per group (N=4). Histamine was observed for 30 minutes after administration and IL31 for 90 minutes after administration. Data are presented as the mean ± standard error (SE), ns: not significant.
Supplementary material Ishikawa et al.
